# Early population-level impact of *Helicobacter pylori* eradication on gastric cancer mortality in Japan: a counterfactual analysis of short-term divergence

**DOI:** 10.1186/s12963-026-00487-0

**Published:** 2026-05-25

**Authors:** Akiko Kowada

**Affiliations:** 1 Department of Occupational Health, Kitasato University Graduate School of Medical Sciences, 1-15-1 Kitasato, Minami-ku, Sagamihara, Kanagawa 252-0373 Japan; 2 Advanced Research Promotion Center, Health Sciences University of Hokkaido, 1757 Kanazawa, Tobetsu‑cho, Ishikari-gun, Hokkaido 061‑0293 Japan

**Keywords:** *Helicobacter pylori*, Eradication, Gastric cancer, Mortality, Counterfactual analysis

## Abstract

**Background:**

Gastric cancer has historically been driven by long‑standing *Helicobacter pylori* infection. The nationwide expansion of *H. pylori* eradication therapy beginning in 2013 created a unique opportunity to evaluate its population‑level impact on gastric cancer mortality. However, short‑term mortality trends following eradication are difficult to interpret because they reflect overlapping influences of ageing, cohort replacement, and cumulative infection history. This study aimed to provide a model‑based, population‑level assessment of the early impact of eradication during the first decade of nationwide implementation.

**Methods:**

We applied a two‑layer analytic framework consisting of a counterfactual analysis comparing observed mortality during 2013–2021 with expected mortality had eradication uptake remained at pre‑2013 levels, combined with a structured state‑transition (Markov) model with time‑dependent parameters. To estimate annual gastric cancer deaths prevented and the proportion of mortality reduction attributable to eradication, the model integrated age‑specific biological hazard, cumulative infection history, cohort‑specific *H. pylori* prevalence, and annual changes in eradication uptake.

**Results:**

Observed gastric cancer deaths declined from 48,632 in 2013 to 41,624 in 2021, whereas counterfactual gastric cancer deaths declined more modestly, from 49,779 to 49,453. The divergence between observed and counterfactual deaths steadily widened from 1,147 in 2013 to 7,829 in 2021. Model‑based estimates indicated that eradication prevented 6,461 gastric cancer deaths during 2013–2021, with annual deaths prevented increasing from 165 in 2015 to 1,604 in 2021, particularly among adults aged 60–79, who showed the most pronounced early benefit reflecting cumulative infection history and real-world uptake patterns.

**Conclusions:**

The early population‑level impact of *H. pylori* eradication is consistent with a 16% reduction in gastric cancer deaths by 2021. These findings provide real‑world insight into how primary prevention can shape short‑term national cancer trends. This approach offers a quantitative framework to inform future prevention strategies in high‑prevalence settings seeking to evaluate early implementation effects.

**Graphical Abstract:**

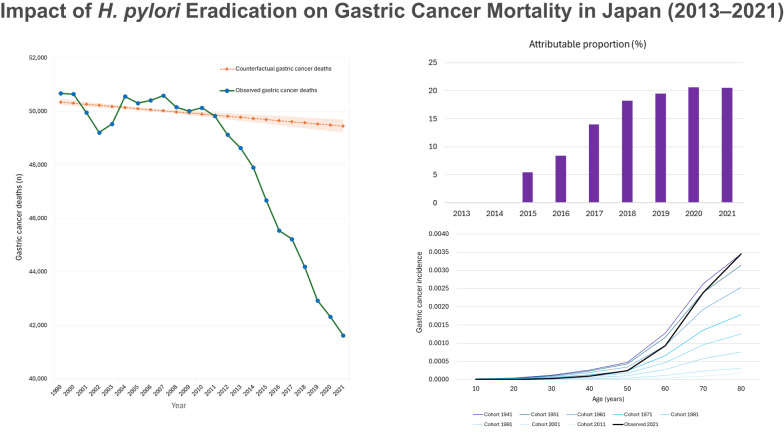

**Supplementary Information:**

The online version contains supplementary material available at 10.1186/s12963-026-00487-0.

## Introduction

Gastric cancer remains a major public health concern in Japan, where it continues to be the third leading cause of cancer‑related deaths [1]. *Helicobacter pylori* infection is the predominant etiologic factor [2], accounting for nearly all cases nationwide [3]. Although *H. pylori* infection prevalence has declined across successive birth cohorts, Japan retains a unique epidemiologic structure in which older adults carry high lifetime exposure [4], shaping national gastric cancer trends for decades.

In 2013, Japan became the first country to introduce nationwide insurance coverage for *H. pylori* eradication therapy for chronic gastritis [5]. This policy led to a rapid expansion of eradication uptake and created a rare opportunity to evaluate the early, population‑level impact of eradication in a real‑world setting (Figure S1). Previous modeling work has shown that population‑based *H. pylori* eradication produces substantial lifetime reductions in gastric cancer mortality and is cost‑saving across all age groups [6]. Subsequent study identified younger age as the optimal timing for population‑based screening and eradication [7]. These findings underscore the importance of evaluating eradication strategies within a time‑dependent, cohort‑structured framework. However, interpreting short‑term mortality changes is challenging because observed trends reflect multiple overlapping forces, including ageing, cumulative infection history [8], cohort replacement [9], and evolving clinical practice [10]. Conventional before–after comparisons or trend‑based analyses cannot disentangle these structural determinants and therefore cannot isolate the specific contribution of eradication [11]. A counterfactual analysis is therefore needed to quantify the short‑term divergence in gastric cancer deaths in the absence of eradication.

To address this complexity, the present study employs a two‑layer analytic framework. First, a structured state‑transition (Markov) model with time‑dependent parameters reconstructs etiologic incidence by integrating age‑specific biological hazard, duration‑related exposure, and cohort‑specific *H. pylori* prevalence. This modeling layer provides a biologically coherent representation of the pathway linking *H. pylori* infection to gastric cancer. Second, a counterfactual analysis [12] compares observed mortality with an expected trajectory representing a scenario in which eradication uptake remained at pre‑2013 levels. This design enables a model-based, population‑level assessment of the early impact of eradication during its initial decade of nationwide implementation.

Using this combined modeling and counterfactual approach, the study aims to quantify the proportion of early reductions in gastric cancer mortality attributable to *H. pylori* eradication within the overall burden of gastric cancer deaths in Japan between 2013 and 2021. Clarifying these short‑term population‑level effects helps illustrate how primary prevention can begin to influence national cancer trends within a relatively brief period.

## Methods

### Study design and overview

**Fig. 1:**
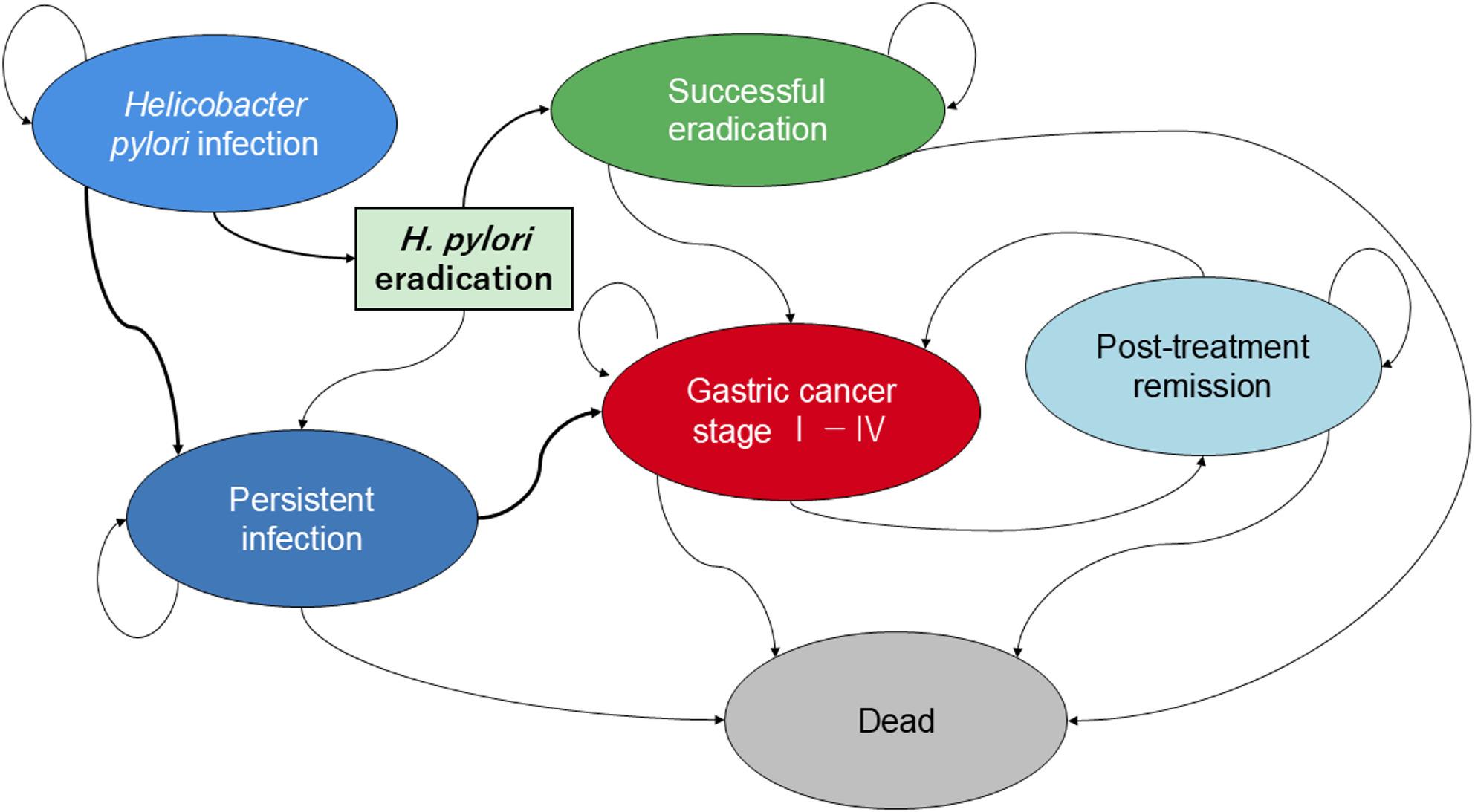
Clinical pathway and outcomes of *H. pylori* infection and eradication. This diagram depicts the natural history of *H. pylori* infection and the potential clinical outcomes following eradication therapy. Initial infection may persist or be cleared through eradication treatment. Persistent infection increases the risk of developing gastric cancer, which can progress to death or transition to post‑treatment remission. Successful eradication reduces the subsequent risk of gastric cancer. Arrows represent possible transitions between health states, including recurrence after remission. Colors indicate distinct clinical phases: blue for natural history, green for intervention, red for cancer progression, light blue for remission, and grey for death

We developed a structured state‑transition (Markov) model with time‑dependent parameters (Fig. 1) [12, 13] to evaluate the population-level impact of Japan’s nationwide *H. pylori* eradication strategy implemented under the universal health insurance system. The analysis covered 2013–2021, corresponding to the period during which eradication uptake expanded rapidly following insurance coverage. Two scenarios were compared: the observed mortality reflecting real-world eradication uptake, and a counterfactual mortality trajectory representing expected mortality if eradication uptake had remained at pre-2013 levels. Comparing these scenarios isolates the contribution of eradication to short‑term changes in gastric cancer mortality while preserving etiologic coherence with the established biology and epidemiology of *H. pylori*–related carcinogenesis.

### Model structure

#### Multiplicative reconstruction of age‑specific gastric cancer incidence

To capture the structural determinants of gastric cancer risk, we reconstructed age-specific incidence using a multiplicative formulation integrating four components [8, 9]: (1) Baseline incidence was defined as the gastric cancer incidence at age 40 in 2021, obtained from the national cancer registry [1]. This served as the reference scale. (2) Age-specific ageing factor was derived from 2021 incidence data to represent biological ageing effects independent of cohort exposure. (3) Duration-related historical exposure factor was estimated by comparing age-specific incidence in 2021 with incidence patterns from earlier high-burden periods. This factor captures cumulative historical exposure not explained by ageing alone. (4) Age- and cohort-specific *H. pylori* prevalence was obtained from a systematic review and meta-regression of 170,752 individuals [4]. These prevalence estimates were mapped to each age and calendar year.

The final incidence formulation was:

$$\begin{aligned}\:{\mathrm{Incidence}}_{a,t}&=\mathrm{Baseline}\times\:{\mathrm{Ageing}}_{a} \\ & \quad\times\:{\mathrm{Duration}}_{a}\times\:{\mathrm{Prevalence}}_{a,t}\end{aligned}$$

This formulation incorporates cumulative infection history by accounting for age, duration of infection, and cohort‑specific exposure. The separation of Ageing and Duration components and the cohort‑specific incidence patterns are illustrated in Supplementary Figure S2.

### Mortality dynamics

#### Counterfactual mortality projection

To quantify the contribution of eradication uptake, we constructed a counterfactual mortality projection [11] representing the expected mortality trend if eradication uptake had remained at its 2013 level. All other structural determinants such as ageing, duration, cohort effects, and case-fatality were held constant to isolate the effect of eradication. Counterfactual gastric cancer deaths were projected using a linear regression model estimated by ordinary least squares (OLS) and fitted to national mortality statistics from 1999 to 2012. The model included calendar year as a continuous predictor and indicator terms for the post‑policy period to allow for level and slope changes.

The fitted model was:

$$\begin{aligned}\:\text{Gastric cancer d}\mathrm{eath}{\mathrm{s}}_{\mathrm{t}}&=131922.55-40.81\mathrm{Yea}{\mathrm{r}}_{\mathrm{t}} \\ & \quad+1831282.33\mathrm{Pos}{\mathrm{t}}_{\mathrm{t}}-910.23\mathrm{TimePos}{\mathrm{t}}_{\mathrm{t}}+{\epsilon\:}_{t}\end{aligned}$$

where Post indicates years ≥ 2013 and TimePost counts years since 2013.

The model showed good fit to pre‑policy data (*R* = 0.9948, R² = 0.9896), with residuals displaying no systematic patterns.　 ANOVA indicated that the overall regression model was highly significant (F = 699, *p* < 1 × 10⁻¹⁵).

Out‑of‑sample performance was assessed by comparing projected values for 2013–2021 with observed deaths; the projections remained within the expected uncertainty range and showed no structural deviations.

#### Difference between observed and counterfactual gastric cancer deaths

Difference between observed and counterfactual gastric cancer deaths was defined as:

$$\begin{aligned}\:{\mathrm{Difference}}_{t}&={\text{Gastric cancer deaths}}_{\mathrm{counterfactual},t}\\ & \quad-{\text{Gastric cancer deaths}}_{\mathrm{observed},t}\end{aligned}$$

This difference reflects the divergence between observed and expected deaths.

#### Estimated gastric cancer deaths prevented by eradication

Deaths prevented were estimated by comparing two strategies within a structured state‑transition (Markov) model with time‑dependent parameters: (1) continuation of standard gastric cancer screening and (2) implementation of *H. pylori* eradication in addition to standard gastric cancer screening. This comparison yielded the reduction in gastric cancer deaths attributable to eradication.　Annual eradication counts for 2016–2021 were estimated using nonpublic aggregated data obtained under confidentiality agreements. These data include year‑specific information sufficient to estimate national eradication volumes.

#### **Attributable proportion of gastric cancer deaths prevented by eradication**

**Fig. 2:**
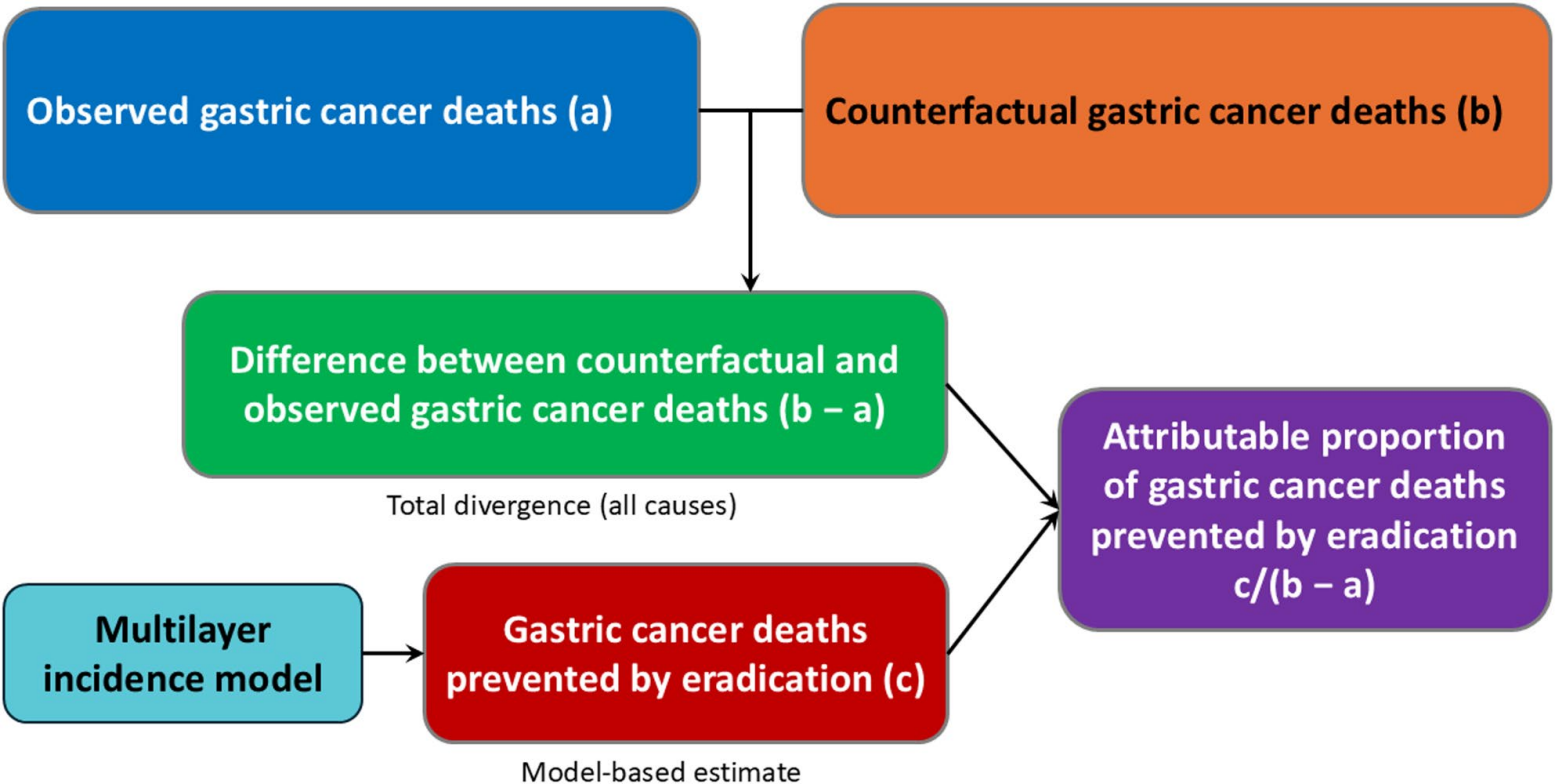
Conceptual framework for estimating the impact of *H. pylori* eradication on gastric cancer mortality. Observed gastric cancer deaths (2013–2021) were compared with counterfactual deaths projected from mortality trends calibrated to national statistics from 1999–2012. The mortality difference (b − a) represents the divergence between counterfactual and observed deaths in the absence of increased eradication uptake. Gastric cancer deaths prevented by eradication (c) quantify the portion of this divergence attributable to eradication. The attributable proportion (c / (b − a)) expresses the relative contribution of eradication within the total mortality divergence

Attributable proportion of gastric cancer deaths prevented by eradication was calculated as:

$$\:{\mathrm{Proportion}}_{t}=\frac{{\text{Deaths prevented by eradication}}_{t}}{{\mathrm{Difference}}_{t}}$$

where Difference represents the counterfactual minus observed difference in gastric cancer deaths in year *t*, indicating the share explained by eradication (Fig. 2).　 This modeling framework also enabled estimation of year-specific gastric cancer deaths prevented by eradication from 2013 to 2021.

### Data inputs

Age-specific mortality, population structure, and gastric cancer incidence were obtained from national cancer statistics [1] and vital statistics [14]. Annual eradication counts were derived from published literature [15] and non-public regional datasets (Figure S1). Model parameters related to screening adherence [16], endoscopy accuracy [17], eradication compliance [18], eradication success [18], and gastric cancer risk reduction with eradication [19] were obtained from published studies (Table S1). Parameters were classified as either empirically derived or assumption‑based. Assumption‑based parameters were subjected to deterministic sensitivity analysis to assess their influence on model outputs.

Epidemiologic parameters related to gastric cancer progression were derived from national cancer statistics [20] and from prior long‑term modeling work evaluating the cumulative lifetime impact of eradication on gastric cancer mortality [6]. Age-specific stage distributions for non‑screen‑detected gastric cancers were incorporated using national cancer statistics [20] (Table S2). These age-specific distributions are consistent with evidence showing that younger patients typically present with more advanced disease and aggressive tumor biology [21, 22].

All data sources were harmonized to produce internally consistent age- and year-specific inputs. All modeling analyses were conducted using TreeAge Pro 2026 (TreeAge Software, Williamstown, MA).

### Sensitivity analysis

To assess parameter uncertainty, we conducted one-way sensitivity analyses on key model inputs. We varied gastric cancer risk reduction after eradication (0.40–0.72), the adherence to endoscopy after eradication among individuals aged ≥ 50 years (0.10–0.50), the adherence to endoscopy among screened individuals (0.10–0.50), and the eradication success rates (0.80–0.99), based on plausible ranges from the literature and assumptions.

### Model validation

The counterfactual mortality projection was calibrated using observed national gastric cancer mortality from 1999 to 2012. A linear regression model, estimated via ordinary least squares (OLS), was fitted to these pre‑intervention data. Model diagnostics (residual patterns and goodness‑of‑fit statistics) confirmed that the pre‑2013 trend was stable and well‑approximated by a linear function. Extrapolated values for 2013–2021 showed no structural inconsistencies relative to the calibrated trend.

For the structured state‑transition model, internal validity was assessed by confirming that age‑specific transition probabilities, infection‑related hazard functions, and cohort‑specific *H. pylori* prevalence patterns were consistent with published epidemiologic estimates. External validity was evaluated by comparing model‑generated mortality trajectories under the no‑eradication scenario with　observed pre‑2013 mortality trends. Sensitivity analyses varying key assumptions demonstrated that the direction and magnitude of estimated mortality reductions were robust across plausible parameter ranges.

## Results

### Trends in observed and counterfactual mortality

**Fig. 3:**
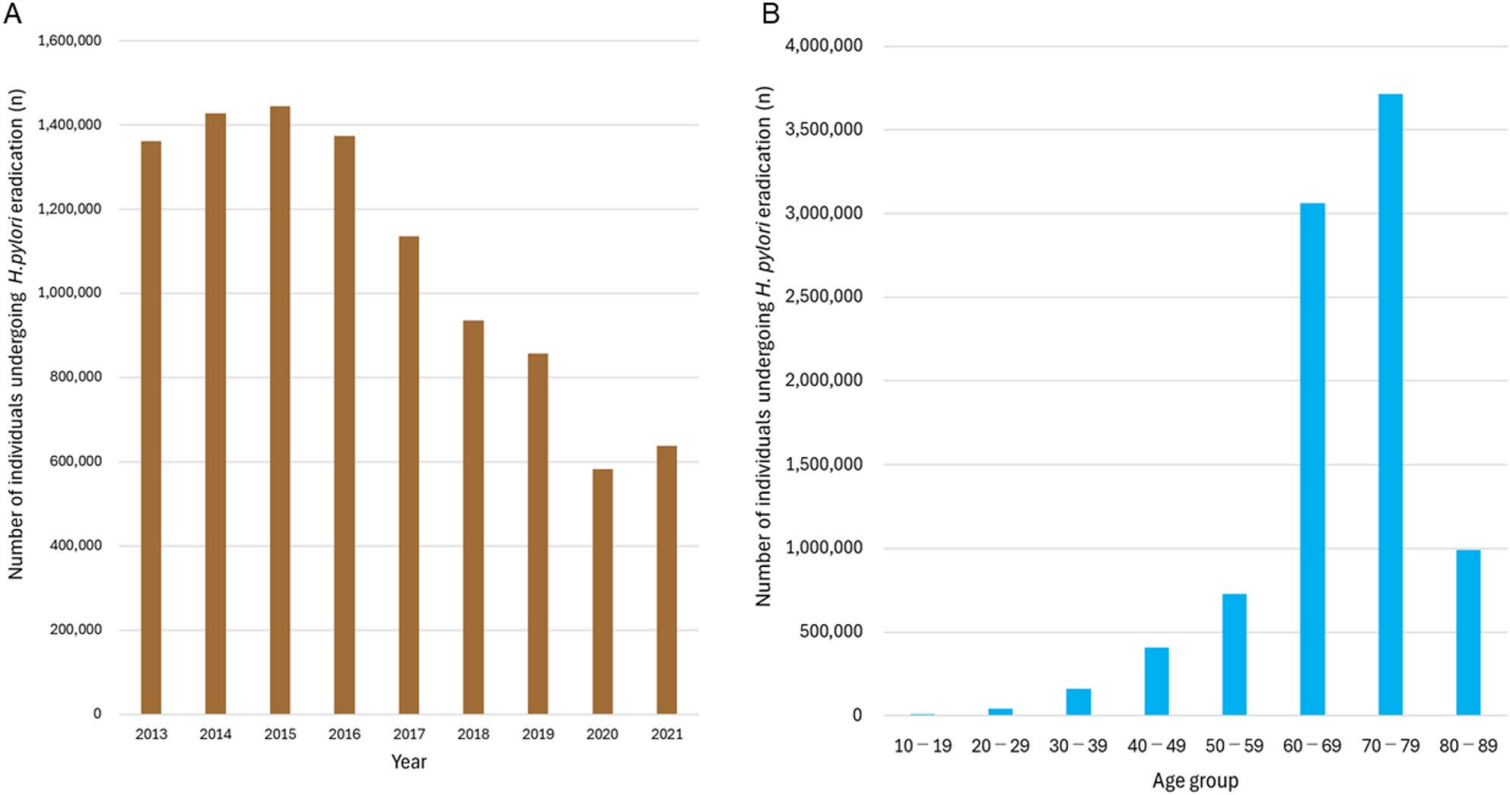
Annual and age-specific numbers of individuals undergoing *H. pylori* eradication in Japan. **A**. Annual number of individuals undergoing *H. pylori* eradication in Japan, 2013–2021. **B**. Number of individuals undergoing *H. pylori* eradication, by age group

Annual trends in the number of individuals undergoing *H. pylori* eradication are shown in Fig. 3A, demonstrating a rapid increase following the introduction of insurance coverage in 2013, followed by a gradual decline, a marked drop during the COVID-19 pandemic, and recent plateau (Figure S1). The majority of eradication uptake occurred among adults aged 60–79 years, consistent with the observed concentration of gastric cancer mortality reductions in this age group (Fig. 3B).

**Fig. 4:**
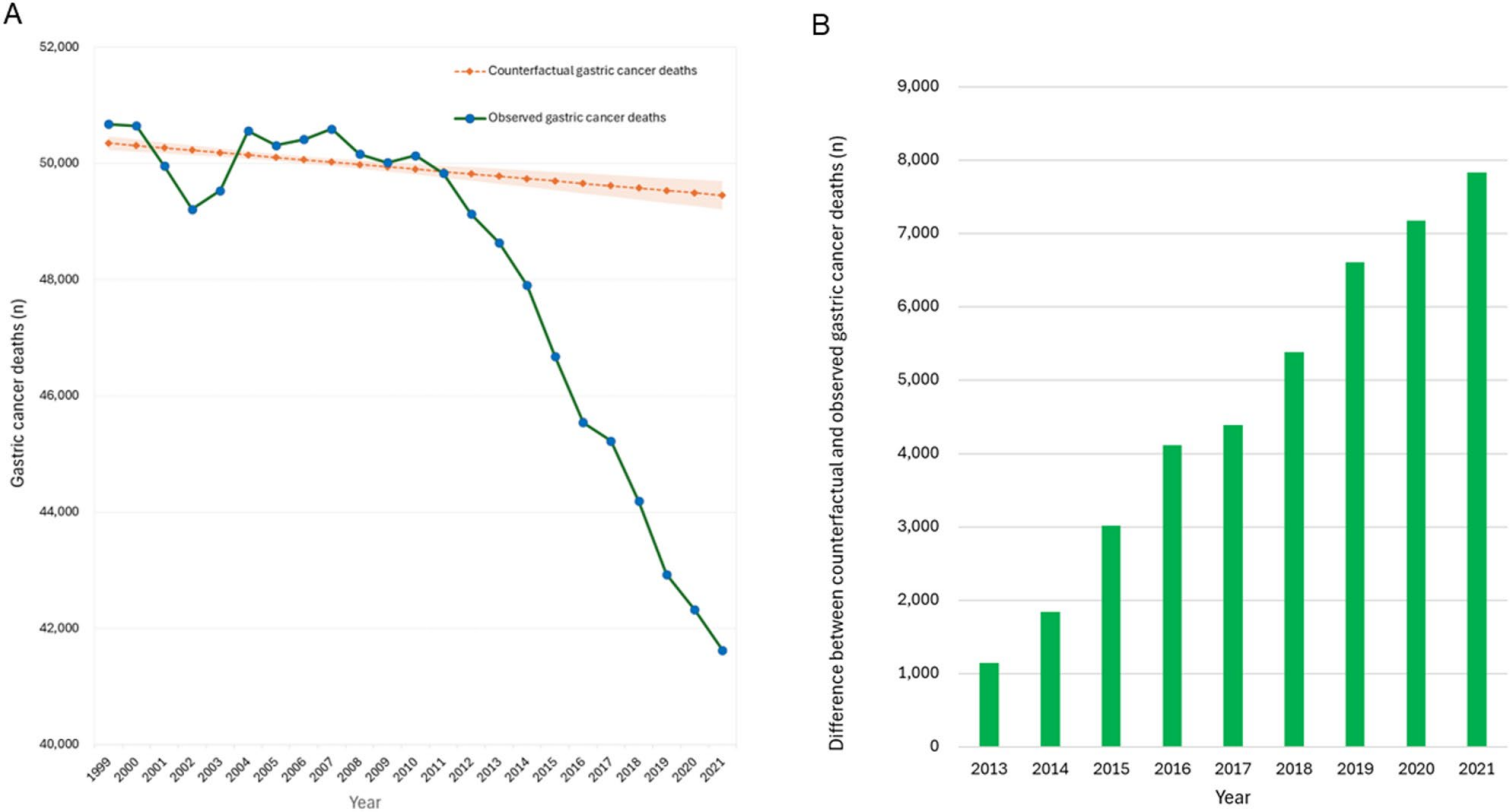
Observed and counterfactual gastric cancer deaths and their annual differences in Japan. **A**. Observed and counterfactual gastric cancer deaths, 2013–2021. **B**. Annual difference between counterfactual and observed gastric cancer deaths

Observed gastric cancer deaths decreased steadily from 48,632 in 2013 to 41,624 in 2021 (Table 1; Fig. 4A). In contrast, the counterfactual mortality trajectory—representing expected mortality if eradication uptake had remained at pre-2013 levels—declined more slowly, from 49,779 deaths in 2013 to 49,453 deaths in 2021 (Table 1; Fig. 4A). The divergence between observed and counterfactual gastric cancer deaths widened steadily, increasing from 1,147 in 2013 to 7,829 in 2021 (Table 1; Fig. 4B). This expanding difference reflects the cumulative impact of increased eradication uptake on national mortality patterns.

**Table 1:** Annual differences between observed and counterfactual gastric cancer deaths and deaths prevented by *H. pylori* eradication, 2013–2021

Year	Observed gastric cancer deaths (a)	Counterfactual gastric cancer deaths (b)	Difference in gastric cancer deaths (b-a)	Deaths prevented by eradication (c)	Attributable proportion (%)
2013	48,632	49,779	1,147	0	0
2014	47,904	49,739	1,835	0	0
2015	46,681	49,698	3,017	165	5
2016	45,546	49,657	4,111	344	8
2017	45,227	49,616	4,389	612	14
2018	44,192	49,575	5,383	977	18
2019	42,931	49,534	6,603	1,283	19
2020	42,319	49,494	7,175	1,475	21
2021	41,624	49,453	7,829	1,604	20
Total	405,056	446,545	41,489	6,461	16

Attributable proportion (%) was calculated as c / (b − a)

### Annual gastric cancer deaths prevented by eradication

**Fig. 5:**
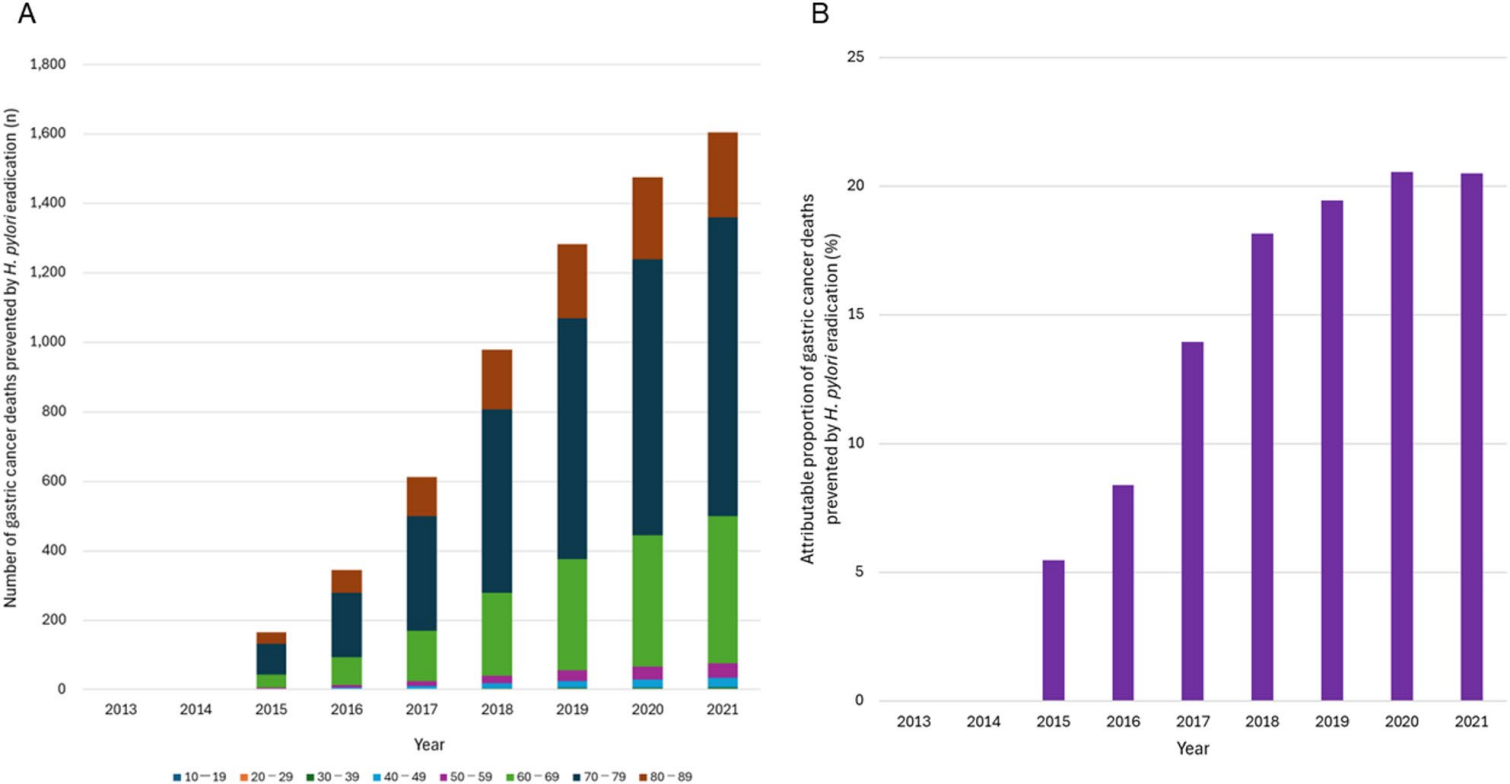
Age-specific and annual impact of *H. pylori* eradication on gastric cancer mortality in Japan. **A**. Number of gastric cancer deaths prevented by *H. pylori* eradication, by age group and year. **B**. Attributable proportion of gastric cancer deaths prevented by *H. pylori* eradication, by year

Model-based estimates indicated that the number of gastric cancer deaths prevented by eradication increased gradually throughout the study period. Deaths prevented rose from 165 in 2015—when the earliest measurable effects of eradication became detectable—to 1,604 in 2021, with a cumulative total of 6,461 deaths prevented during 2013–2021 (Table 1; Fig. 5A).

The largest annual reductions were observed among adults aged 60–79, consistent with both the age distribution of eradication uptake and the cohort-specific infection histories that shape gastric cancer risk　(Fig. 5A).

### Attributable proportion of gastric cancer deaths prevented by eradication

The attributable proportion of gastric cancer deaths prevented by eradication increased steadily over time. This proportion rose from 5% in 2015 to 20% in 2021 (Table 1; Fig. 5B).

This upward trend indicates that eradication has become an increasingly important contributor to Japan’s declining gastric cancer mortality, even within the relatively short nine-year period following nationwide insurance coverage.

### Sensitivity analysis

**Fig. 6:**
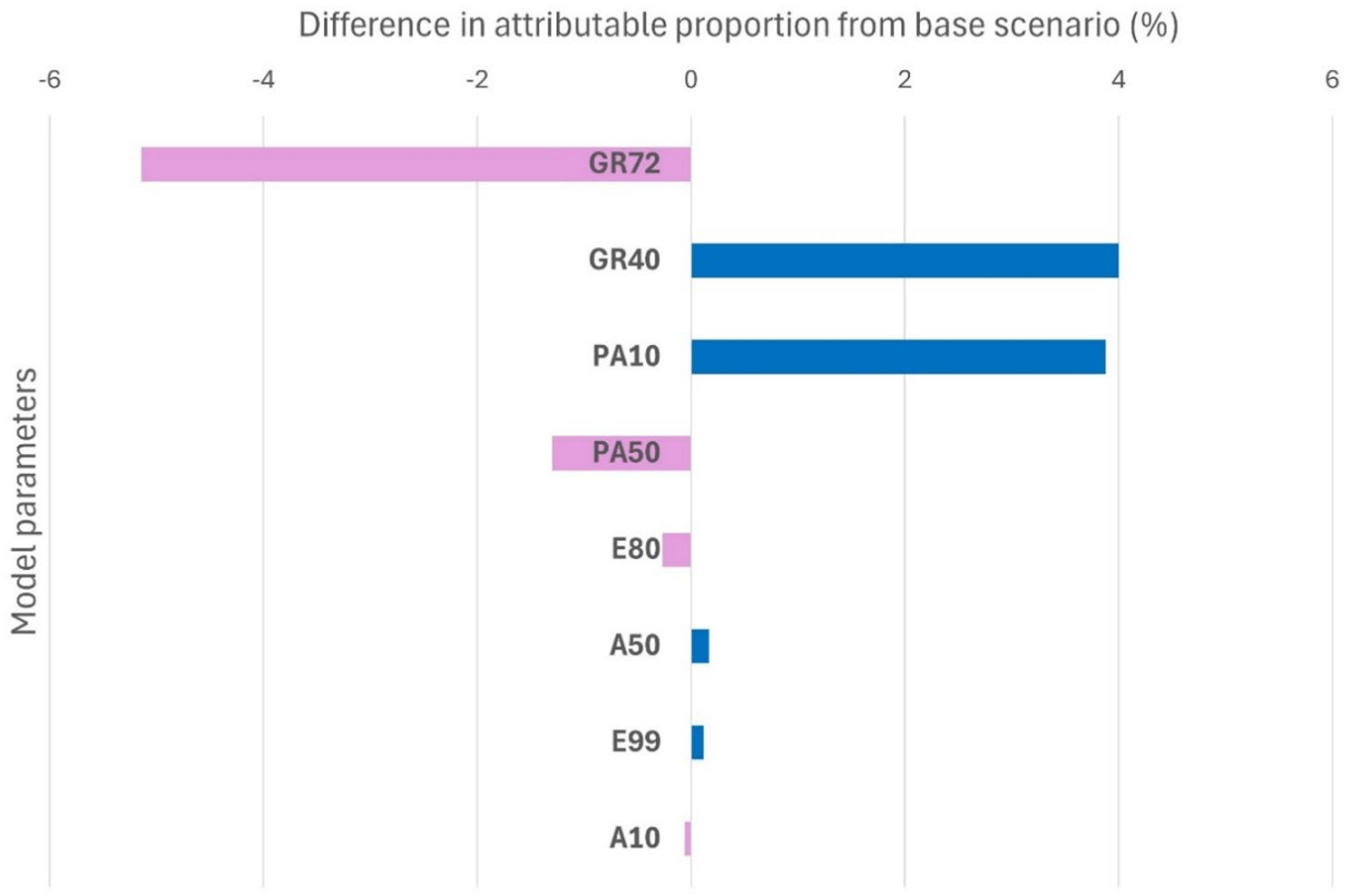
Sensitivity analysis of the attributable proportion of gastric cancer deaths prevented by *H. pylori* eradication. Bars indicate the change in the attributable proportion (%) when varying key model parameters relative to the base scenario. Parameter definitions: GR72 = gastric cancer risk reduction after eradication of 0.72, GR40 = gastric cancer risk reduction after eradication of 0.40, PA10 = adherence rate of endoscopy after eradication (age ≥ 50) of 0.10, PA50 = adherence rate of endoscopy after eradication (age ≥ 50) of 0.50, E80 = eradication success rate of 0.80; E99 = eradication success rate of 0.80, A50 = adherence rate to endoscopy among screened individuals (age ≥ 50) of 0.50, E99 = eradication success rate of 0.80; E99 = eradication success rate of 0.99, A10 = adherence rate to endoscopy among screened individuals (age ≥ 50) of 0.10

One-way sensitivity analyses showed that the estimated number of gastric cancer deaths prevented was robust to wide variations in key parameters (Fig. 6). When gastric cancer risk reduction with eradication was varied from 0.40 to 0.72, the corresponding number of deaths prevented decreased from 8,121 to 4,330 (Table S3, Figure S3). Varying the adherence to endoscopy after eradication from 0.10 to 0.50 yielded estimates between 5,921 and 8,069 deaths prevented. Changes in adherence to endoscopy among screened individuals and eradication success rates had only modest effects on the estimates. In all scenarios, eradication was associated with a substantial reduction in gastric cancer mortality.

### Summary of early population-level impact of *H. pylori* eradication

Together, these findings demonstrate that:

- gastric cancer mortality in Japan is declining faster than expected under a no-expansion counterfactual.
- eradication has prevented more than 6,400 deaths.
- the contribution of eradication is increasing each year.
- the strongest effects are concentrated in middle-aged and older adults, reflecting cumulative infection history and real-world uptake patterns.
- the results of sensitivity analyses are consistent with a substantial reduction in gastric cancer mortality.

These results suggest that *H. pylori* eradication is already contributing to observable changes in national gastric cancer trends during the early phase of its nationwide implementation.

## Discussion

### Interpretation of findings

Gastric cancer mortality in Japan began declining more rapidly than expected around 2015, and the divergence between observed and counterfactual mortality widened steadily through 2021. Although cancer prevention is often assumed to require long time horizons, these findings indicate that in high‑prevalence settings, measurable reductions can emerge within a decade.

The largest early effects were observed among adults aged 60–79, reflecting both the age distribution of eradication uptake and cumulative infection histories that shape gastric cancer risk. In addition to risk reduction following eradication, individuals aged ≥ 50 years commonly undergo follow‑up endoscopy, which may contribute to earlier diagnosis, stage shift, and improved case fatality. These diagnostic and prognostic mechanisms likely contributed to the early decline in mortality.

The number of deaths prevented by eradication represents one component of the overall mortality decline. Early estimates were sensitive to adherence to post‑eradication endoscopy and gastric cancer risk reduction after eradication. Clarithromycin resistance may modestly reduce eradication success; however, sensitivity analyses showed that the direction and magnitude of mortality reduction remained robust across plausible ranges. Given that screening uptake had only a minor impact on mortality trends in our analyses, the divergence between observed and counterfactual gastric cancer deaths is substantially explained by *H. pylori* eradication.

### Structural epidemiologic implications

This study illustrates the value of combining a structured incidence model with a counterfactual mortality projection to interpret short‑term changes in gastric cancer mortality. The incidence reconstruction integrates age‑specific biological hazard, cumulative infection history, and cohort‑specific *H. pylori* prevalence, providing a coherent representation of how long‑standing infection shapes population‑level risk. The counterfactual framework isolates the additional mortality reduction associated with increased eradication uptake, separate from background trends such as ageing, cohort replacement, diagnostic intensity, screening, and treatment improvements. This approach enables a clearer interpretation of early mortality changes than conventional before‑after or trend‑based analyses.

### Policy relevance

These findings highlight the potential for primary prevention to influence national cancer trends and provide a quantitative framework for evaluating eradication strategies in other high‑prevalence settings. Japan’s experience further demonstrates that population‑based *H. pylori* eradication can produce measurable reductions in gastric cancer mortality within a relatively short period.

### Comparison with conventional modeling and forecasting approaches

Conventional natural history models often rely on unobservable transition parameters and assumptions about latent disease states. In contrast, the present framework reconstructs incidence directly from observable etiologic determinants, providing a transparent representation of population‑level risk. Forecasting‑based approaches, such as ARIMA or interrupted time‑series regression [23], are useful for projecting future trends but cannot isolate the mortality reduction specifically associated with eradication because they extrapolate past patterns without reconstructing counterfactual disease dynamics. The model‑based counterfactual approach used here offers a more rigorous estimate of the short‑term impact of real‑world eradication efforts.

### Strengths and limitations

This study has several strengths. First, the multilayer gastric cancer incidence model separates age‑specific, duration‑related, and cohort‑specific components of gastric cancer risk, enabling *H. pylori* prevalence‑dependent changes in age‑specific incidence to be detected. This improved resolution in incidence dynamics enhances the precision of estimates of gastric cancer deaths prevented. Second, the Markov cohort framework represents year‑to‑year transitions explicitly, capturing how risk evolves as cohorts age and enabling detailed age‑ and cohort‑specific analyses. Third, the analysis was anchored in a screening‑present context, which minimizes the influence of changes in screening practices on mortality trends. By holding the screening environment constant within the model, the effect of *H. pylori* eradication can be isolated more clearly, reducing potential bias arising from fluctuations in detection rates or stage distribution. Fourth, the model reconstructs incidence from observable determinants rather than relying on unobservable transition parameters, providing transparent inference in a setting where comprehensive real‑world eradication data are not available. Finally, extensive sensitivity analyses—covering alternative assumptions for risk reduction after eradication, adherence to endoscopy, eradication success, and post‑eradication follow‑up—demonstrate that the findings are robust across a wide range of plausible scenarios.

Several limitations should also be acknowledged. Real-world data on *H. pylori* eradication in Japan remain limited; nationally representative information on uptake, adherence, and eradication success is not systematically collected, requiring reliance on published ranges rather than individual-level data. The model does not explicitly represent all biological or clinical pathways linking eradication to mortality, and therefore captures only the dominant mechanisms reflected in population-level trends. In addition, the counterfactual projection assumes that pre-2013 mortality trends would have continued under relatively stable screening and clinical practice patterns. Although supported by historical data, unmeasured changes in diagnostic or treatment practices could influence the estimated counterfactual trajectory.

### Generalizability

The findings may be informative for other high‑prevalence countries considering population‑based *H. pylori* eradication, particularly in illustrating how eradication‑driven removal of infection can produce short‑term reductions in gastric cancer mortality. The magnitude and timing of these reductions will depend on local infection prevalence, the age distribution of eradication uptake, background screening practices, and access to treatment. Although these contextual factors vary across settings, the modeling framework provides a transferable structure for reconstructing incidence dynamics and estimating the potential impact of eradication strategies using country‑specific data.

### Future directions

Future work could incorporate individual‑level data on eradication uptake, adherence to follow‑up endoscopy, and eradication success to refine estimates of short‑term mortality effects. Linking eradication records with cancer registry data would enable more detailed evaluation of stage distribution and case fatality. As screening practices evolve, integrating dynamic screening uptake into the model may further improve projections of long‑term population impact. In addition, *H. pylori* prevalence is declining rapidly in successive birth cohorts, creating a demographic shift in which younger adults enter adulthood with historically low infection rates. In this context of both falling prevalence and rapidly decreasing gastric cancer incidence, the relative contribution of eradication in younger cohorts is expected to become increasingly important. Because these generations carry the greatest remaining lifetime risk to avert, the impact of eradication is likely to grow with each successive cohort. Modeling approaches that incorporate this demographic transition are essential for anticipating how eradication in a low‑prevalence era may shape the next phase of gastric cancer reduction.

## Conclusion

Population‑based *H. pylori* eradication in Japan has been associated with measurable reductions in gastric cancer mortality within a decade of expanded coverage. Notably, the 60–79‑year age group accounted for the majority of prevented deaths, indicating that eradication remains highly effective even when implemented later in life. Beyond these short‑term gains, declining infection prevalence in younger cohorts indicates a transition toward preventing transmission to the next generation. These findings demonstrate that primary prevention can alter national cancer trajectories for all generations. As infection prevalence continues to fall in successive birth cohorts, the strategic importance of early‑life eradication will only grow. Our results provide a framework for evaluating eradication policies in other settings undergoing similar demographic transitions and underscore the need to integrate cohort‑based primary prevention into future cancer control strategies.

## Supplementary Information

Supplementary material 1.

## Data Availability

No datasets were generated or analysed during the current study.

## References

[1] Cancer Information Service, National Cancer Center, Japan. Cancer registry and statistics. Available at: https://ganjoho.jp/reg_stat/statistics/stat/cancer/5_stomach.html. Accessed January 30, 2026.

[2] IARC Working Group. Population-based *Helicobacter pylori* screen-and-treat strategies for gastric cancer prevention: guidance on implementation. In: Park JY, editor. IARC Working Group Report No. 12. Lyon. France: International Agency for Research on Cancer; 2025. 40601795

[3] Matsuo T, Ito M, Takata S, et al. Low prevalence of *Helicobacter pylori*-negative gastric cancer among Japanese. Helicobacter. 2011;16:415–9. 10.1111/j.1523-5378.2011.00889.x. 22059391 10.1111/j.1523-5378.2011.00889.x

[4] Wang C, Nishiyama T, Kikuchi S, et al. Changing trends in the prevalence of *H. pylori* infection in Japan (1908–2003): a systematic review and meta-regression analysis of 170,752 individuals. Sci Rep. 2017;7:15491. 10.1038/s41598-017-15490-7. 29138514 10.1038/s41598-017-15490-7 PMC5686167

[5] Asaka M, Kato M, Sakamoto N. Roadmap to eliminate gastric cancer with *Helicobacter pylori* eradication and consecutive surveillance in Japan. J Gastroenterol. 2014;49(1):1–8. 10.1007/s00535-013-0897-8. 24162382 10.1007/s00535-013-0897-8 PMC3895201

[6] Kowada A, Asaka M. Economic and health impacts of introducing *Helicobacter pylori* eradication strategy into national gastric cancer policy in Japan: a cost-effectiveness analysis. Helicobacter. 2021;26:e12837. 10.1111/hel.12837. 34278663 10.1111/hel.12837 PMC9286640

[7] Kowada A. Cost-Effectiveness of Population-Based Helicobacter pylori Screening With Eradication for Optimal Age of Implementation. Helicobacter. 2024;29(4):e13120. 10.1111/hel.13120. 39138610 10.1111/hel.13120

[8] Anderson RM, May RM. Infectious Diseases of Humans: Dynamics and Control. Oxford: Oxford University Press; 1991.

[9] Clayton D, Schifflers E. Models for temporal variation in cancer rates. I: Age–period–cohort models. Stat Med. 1987;6(4):449–67. 3629047 10.1002/sim.4780060405

[10] Cutler DM, McClellan M. Is technological change in medicine worth it? Health Aff (Millwood). 2001;20(5):11–29. 11558696 10.1377/hlthaff.20.5.11

[11] Rothman KJ, Greenland S, Lash TL. Modern Epidemiology. 3rd ed. Philadelphia: Lippincott Williams & Wilkins; 2008.

[12] Hernán MA, Robins JM. Causal Inference: What If. Boca Raton: Chapman & Hall/CRC; 2020.

[13] Moolgavkar SH, Holford TR, Levy DT, et al. Impact of reduced tobacco smoking on lung cancer mortality in the United States during 1975–2000. Risk Anal. 2009;29:483–97. 10.1111/j.1539-6924.2008.01179.x. 22423009 10.1093/jnci/djs136 PMC3317881

[14] Ministry of Health. Labour and Welfare, Japan. Vital statistics. Available at: https://www.mhlw.go.jp/english/database/db-hw/vs01.html. Accessed January 30, 2026.

[15] Hiroi S, Sugano K, Tanaka S, Kawakami K. Impact of health insurance coverage for *Helicobacter pylori* gastritis on the trends in eradication therapy in Japan: retrospective observational study and simulation study based on real-world data. BMJ Open. 2017;7(7):e015855. 10.1136/bmjopen-2017-015855 28760790 10.1136/bmjopen-2017-015855 PMC5642792

[16] Ministry of Health, Labour and Welfare. Prefectural cancer screening rate 2022. National life foundation survey. Available at: https://www.mhlw.go.jp/toukei/saikin/hw/k-tyosa/k-tyosa22/index.html. Accessed January 30, 2026.

[17] Hamashima C, Okamoto M, Shabana M, et al. Sensitivity of endoscopic screening for gastric cancer by the incidence method. Int J Cancer. 2013;133:653–9. 10.1002/ijc.28065. 23364866 10.1002/ijc.28065

[18] Mori H, Suzuki H, Omata F, et al. Current status of first- and second-line *Helicobacter pylori* eradication therapy in the metropolitan area: a multicenter study with a large number of patients. Ther Adv Gastroenterol. 2019;12:1756284819858511. 10.1177/1756284819858511. 31320930 10.1177/1756284819858511 PMC6611030

[19] Ford AC, Yuan Y, Moayyedi P. *Helicobacter pylori* eradication therapy to prevent gastric cancer: systematic review and meta-analysis. Gut. 2020;69:2113–21. 10.1136/gutjnl-2020-320839. 32205420 10.1136/gutjnl-2020-320839

[20] National Cancer Center Japan. Cancer statistics in Japan 2025. Foundation for Promotion of Cancer Research. 2025. Available at: https://ganjoho.jp/public/qa_links/report/statistics/2025_en.html. Accessed January 30, 2026.

[21] Cheng L, Chen S, Wu W, Zhuang Q, et al. Gastric cancer in young patients: a separate entity with aggressive features and poor prognosis. J Cancer Res Clin Oncol. 2020;146:2937–47. 10.1007/s00432-020-03268-w. . 32451690 10.1007/s00432-020-03268-w PMC11804640

[22] Guan WL, Yuan LP, Yan XL, et al. More attention should be paid to adult gastric cancer patients younger than 35 years old: extremely poor prognosis was found. J Cancer. 2019;10:472–8. 10.7150/jca.27517. 30719142 10.7150/jca.27517 PMC6360302

[23] Bernal JL, Cummins S, Gasparrini A. Interrupted time series regression for the evaluation of public health interventions. Int J Epidemiol. 2017;46(1):348–55. 10.1093/ije/dyw098. 27283160 10.1093/ije/dyw098 PMC5407170

