# Supplementary material for: Early population-level impact of Helicobacter pylori eradication on gastric cancer mortality in Japan: a counterfactual analysis of short-term divergence
Source: Popul Health Metr. 2026 May 25;24:41. doi: 10.1186/s12963-026-00487-0 (PMC13242129; doi:10.1186/s12963-026-00487-0)
Supplement: Supplementary file 1 — Supplementary material 1. [file 12963_2026_487_MOESM1_ESM.pdf]

Supplementary Material – Table of Contents

Table S1. Model parameters, baseline values, and sensitivity analysis ranges used in the Markov cohort model

Table S2. Age-specific stage distribution for non-screen-detected gastric cancers

Table S3. Sensitivity analysis results for gastric cancer deaths prevented by H. pylori eradication

1. Attributable proportion of gastric cancer deaths (%)
2. Number of gastric cancer deaths

Figure S1. Temporal trend in the number of individuals who underwent *H. pylori* eradication in Japan, 2000–2021

Figure S2. Age-specific and cohort-specific gastric cancer incidence patterns used to separate Ageing and Duration components in the multilayer incidence model

Figure S3. Sensitivity analysis of gastric cancer deaths prevented by *H. pylori* eradication

Table S1. Model parameters, baseline values, and sensitivity analysis ranges used in the Markov cohort model

Variable	Baseline	One-way sensitivity analysis range	Distribution	Reference
Proportion of gastric cancer cases attributable to *H. pylori*	0.98	—	β	3
Adherence rate to endoscopy after eradication (age ≥50)	0.20	0.10—0.50	β	Assumption
Adherence rate to current gastric cancer screening (age ≥50)	0.44	—	β	16
Adherence rate to endoscopy among screened individuals	0.196	0.10—0.50	β	16
First-line eradication compliance rate	0.891	—	β	18
First-line eradication success rate	0.901	0.80—0.99	β	18
Second-line eradication compliance rate	0.901	—	β	18
Second-line eradication success rate	0.901	0.80—0.99	β	18
Gastric cancer risk reduction after eradication	0.54	0.40—0.72	β	19
Sensitivity of endoscopy	0.954	—	β	17
Specificity of endoscopy	0.888	—	β	17
Stage distribution for screen-detected gastric cancers	I: 0.898, II: 0.054, III: 0.020, IV: 0.028	—	Dirichlet	20
Stage-specific 5-year survival rate (I-IV)	I: 0.960, II: 0.692, III: 0.419, IV: 0.063	—	β	20

Table S2. Age-specific stage distribution for non-screen-detected gastric cancers

Age group	Stage I	Stage II	Stage III	Stage IV
10–19	0.224	0.074	0.137	0.634
20–29	0.342	0.068	0.124	0.466
30–39	0.500	0.094	0.104	0.302
40–49	0.577	0.098	0.092	0.233
50–59	0.631	0.084	0.094	0.191
60–69	0.637	0.077	0.098	0.188
70–79	0.659	0.077	0.090	0.174
80–89	0.613	0.093	0.096	0.198

Table S3. Sensitivity analysis results for gastric cancer deaths prevented by *H. pylori* eradication

1. Attributable proportion of gastric cancer deaths (%)

Year	Base	GR40	GR72	A50	A10	E99	E80	PA50	PA10
2013	0	0	0	0	0	0	0	0	0
2014	0	0	0	0	0	0	0	0	0
2015	5	5	5	5	5	5	5	5	5
2016	8	9	7	8	8	8	8	9	8
2017	14	16	11	14	14	14	14	16	13
2018	18	22	13	18	18	18	18	22	17
2019	19	24	13	20	19	20	19	24	18
2020	21	27	13	21	20	21	20	27	19
2021	20	27	12	21	20	21	20	27	18

2. Number of gastric cancer deaths

Year	Base	GR40	GR72	A50	A10	E99	E80	PA50	PA10
2013	0	0	0	0	0	0	0	0	0
2014	0	0	0	0	0	0	0	0	0
2015	165	165	165	166	164	165	165	165	165
2016	344	374	306	347	344	345	342	375	334
2017	612	707	489	617	610	614	605	708	579
2018	977	1,186	710	987	974	983	964	1,184	908
2019	1,283	1,614	858	1,297	1,278	1,293	1,261	1,606	1,175
2020	1,475	1,925	898	1,493	1,469	1,488	1,445	1,907	1,330
2021	1,604	2,151	904	1,625	1,597	1,620	1,568	2,125	1,430
Total	6,461	8,121	4,330	6,531	6,435	6,509	6,350	8,069	5,921

Parameter definitions:

GR40 = gastric cancer risk reduction after eradication of 0.40

GR72 = gastric cancer risk reduction after eradication of 0.72

A50 = adherence rate to endoscopy among screened individuals (age ≥50) of 0.50

A10 = adherence rate to endoscopy among screened individuals (age ≥50) of 0.10

E99 = eradication success rate of 0.99

E80 = eradication success rate of 0.80

PA50 = adherence rate of endoscopy after eradication (age ≥50) of 0.50

PA10 = adherence rate of endoscopy after eradication (age ≥50) of 0.10

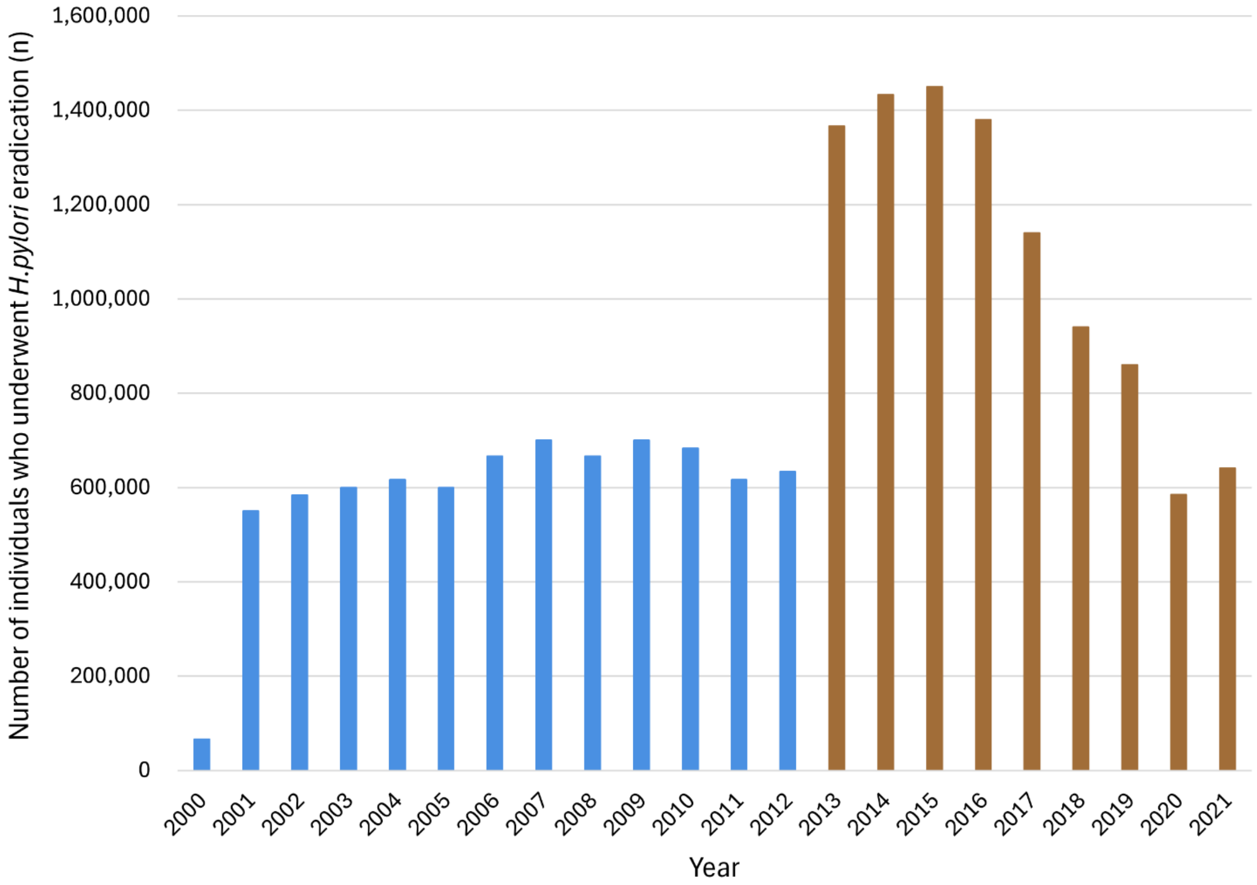

Figure S1. Temporal trend in the number of individuals who underwent *H. pylori* eradication in Japan, 2000–2021

Eradication therapy for peptic ulcer disease has been covered by national insurance since 2000, but the number of individuals receiving eradication remained modest until 2012. The number increased sharply after insurance coverage was expanded to include chronic gastritis in 2013.

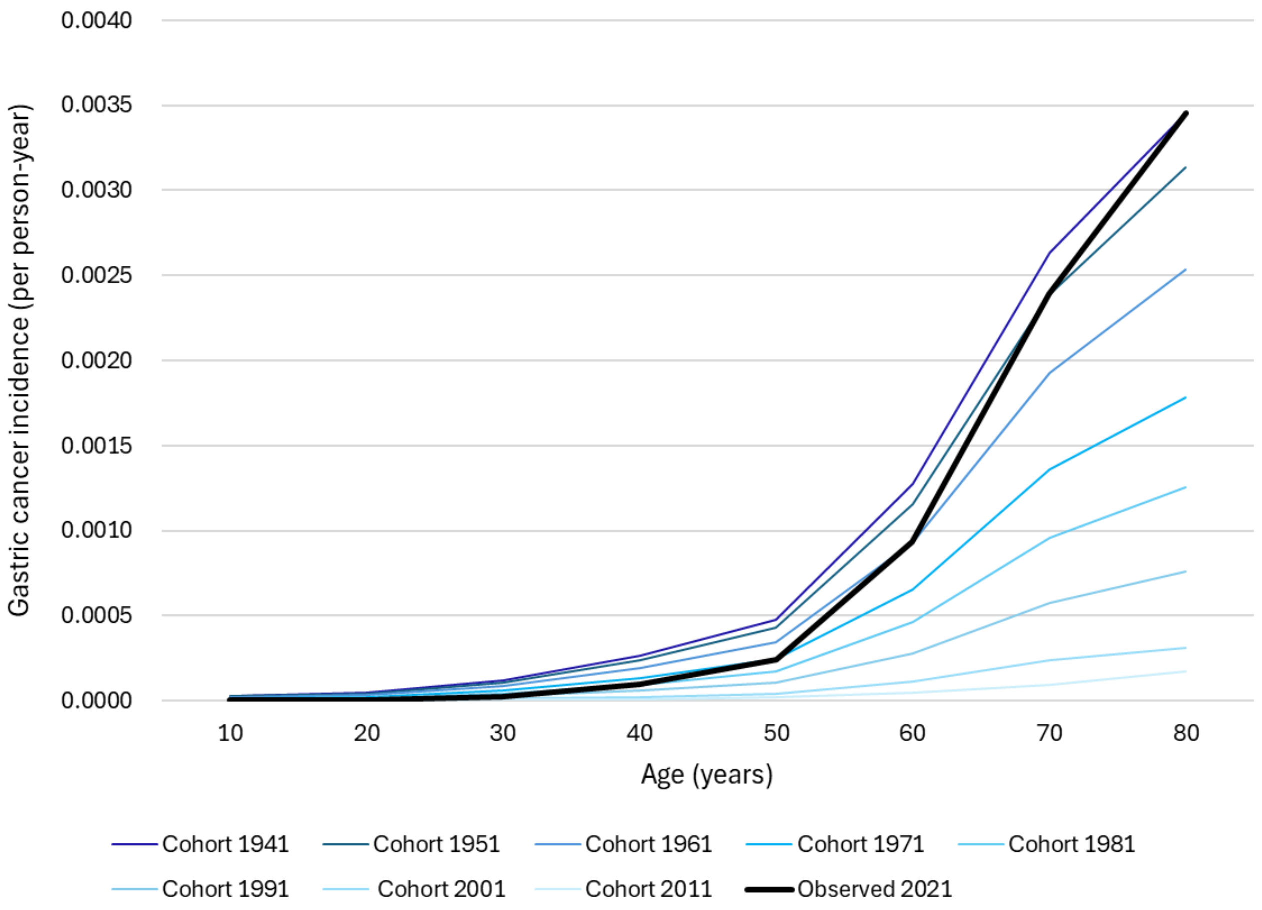

Figure S2. Age-specific and cohort-specific gastric cancer incidence patterns used to separate Ageing and Duration components in the multilayer incidence model

Age-incidence curves are shown for multiple birth cohorts (1941–2011), reconstructed from national cancer registry data. The observed 2021 incidence curve is overlaid for comparison.

The steep age-related increase after age 50 reflects the Ageing component, while systematic differences between cohorts at the same age represent historical exposure patterns captured by the Duration component.

These cohort-specific incidence trajectories illustrate how cumulative infection history and generational exposure differences were incorporated into the incidence formulation.

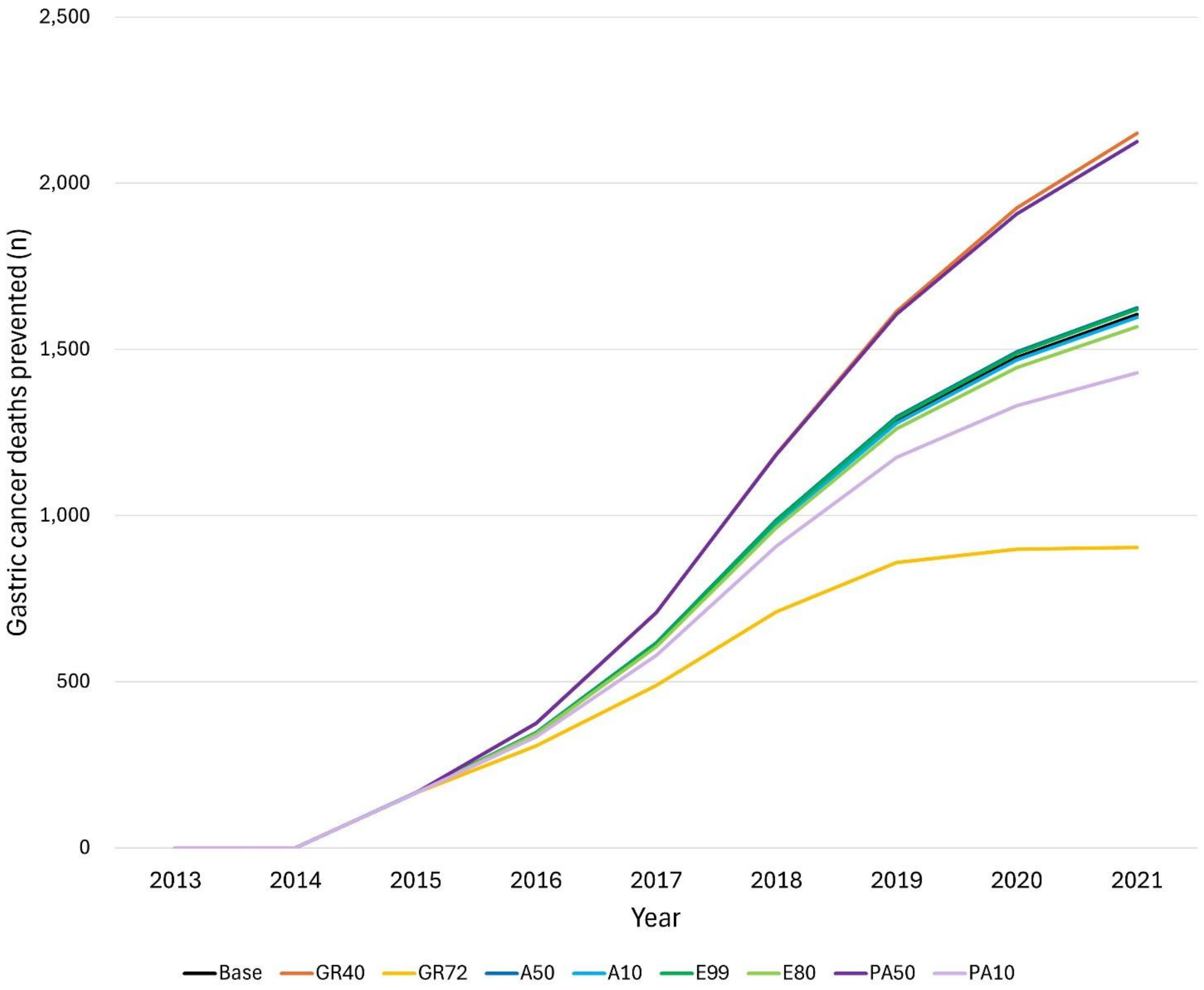

Figure S3. Sensitivity analysis of gastric cancer deaths prevented by *H. pylori* eradication

Annual number of gastric cancer deaths prevented from 2013 to 2021 under different parameter settings in the Markov cohort model. The gastric cancer risk reduction after eradication (GR) had the largest impact on deaths prevented, followed by adherence to post-eradication endoscopy among individuals aged ≥50 years (PA).

Parameter definitions:
GR40 = gastric cancer risk reduction after eradication of 0.40
GR72 = gastric cancer risk reduction after eradication of 0.72
A50 = adherence rate to endoscopy among screened individuals (age ≥50) of 0.50
A10 = adherence rate to endoscopy among screened individuals (age ≥50) of 0.10
E99 = eradication success rate of 0.99
E80 = eradication success rate of 0.80
PA50 = adherence rate of endoscopy after eradication (age ≥50) of 0.50
PA10 = adherence rate of endoscopy after eradication (age ≥50) of 0.10
